# Engaging multi-stakeholder perspectives to identify dementia care research priorities

**DOI:** 10.1186/s41687-021-00325-x

**Published:** 2021-06-22

**Authors:** Neela K. Patel, Sara S. Masoud, Kylie Meyer, Angelica V. Davila, Sheran Rivette, Ashlie A. Glassner, Deborah James, Carole L. White

**Affiliations:** 1 Geriatrics and Supportive Care, Long School of Medicine, UT Health San Antonio, San Antonio, Texas USA; 2 Glenn Biggs Institute for Alzheimer’s and Neurodegenerative Diseases, UT Health San Antonio, San Antonio, Texas USA; 3 Caring for the Caregiver Program, School of Nursing, UT Health San Antonio, San Antonio, Texas USA

## Abstract

**Objectives:**

The purpose of this study was to partner with stakeholders to identify gaps in care for persons living with dementia and their family caregivers and from this list, identify priorities for dementia care research.

**Methods:**

Using a community-engaged research approach, a Stakeholder Advisory Council (SAC) consisting of diverse membership including persons living with dementia and family caregivers was convened. Through our work with the SAC, along with input from the wider network through a symposium, webinars, and an online learning community, gaps in dementia care and a list of topics for dementia care research was generated. This list was reduced to 46 topics for dementia care research and sent to stakeholders (persons living with dementia, family caregivers, and health/social care professionals in dementia care) to be prioritized by rating each of the 46 topics as “Not so important,” “Important,” or “Very important.” Priorities for dementia care were summarized by frequencies and proportions.

**Results:**

A total of 186 participants completed the survey from August through October 2020, including 23 (12.4%) persons living with dementia, 101 (54.3%) family caregivers, and 62 (33.3%) health/social care professionals. Consistent across stakeholder groups was the focus on research on how best to support families following a diagnosis of dementia. Among persons living with dementia, research focused on support for continuing to live in their own homes was ranked as the highest priority, rated by 91.3% as “Very Important”. High priority research areas for family caregivers included interventions to slow cognitive decline (76.3%) as well as non-pharmacological approaches to manage behavioral symptoms (74.7%). The highest priority research topics for health/social care professionals were focused on the diagnosis including benefits of an early diagnosis (71.4%), how best to deliver the diagnosis (70.9%), and supports needed following a diagnosis (78.6%).

**Conclusions:**

This project draws on the strengths of its multi-stakeholder perspective to support patient-centered outcomes research. Findings are intended to inform those who conduct research and those who fund research about which research topics stakeholders believe are most important and thus have greatest potential to improve the quality of life among people living with dementia and their families.

**Supplementary Information:**

The online version contains supplementary material available at 10.1186/s41687-021-00325-x.

## Introduction

There is a growing emphasis on the inclusion of patients and other stakeholders in healthcare research, in order to produce evidence that matters to patients and families and to meaningfully impact dissemination and uptake of findings [1, 2]. A continuum of involvement can be seen in the literature from patients and the public serving as advisors, helping to select the research questions, assisting with the conduct of the study as members of the research team, and even to serving as co-authors on manuscripts [3–5]. Despite the importance of patient-engaged research, persons living with dementia and their families continue to face barriers to active engagement with researchers [6]. Bethell et al. [7] conducted a scoping review to describe the extent of patient/family engagement in research related to dementia and found that, while there is a growing number of research teams that engage persons living with dementia (PLWD) and family caregivers, researchers continue to perceive barriers that include the added costs and time as well as the lack of training required to adapt to a shared decision-making process.

The growing number of families impacted by Alzheimer’s disease and related dementias (ADRD), coupled with the lack of disease-modifying therapies [8], underscores the urgent need for research that addresses priorities in care for families. Bringing the perspective and expertise of families who have the lived experience of dementia to our research is important to the creation of evidence that matters and is beneficial to patients and families [9]. What is important to families often contrasts with professional perspectives; interventions will be impactful only when they make a difference on patient- and caregiver-reported outcomes [10]. Yet, a recent review examining family-centered dementia care research reports limited conceptualization in the literature around family-centered research [11]. The unique perspectives of stakeholders, including those impacted personally by dementia and social/health care professionals as well as researchers who provide care for families impacted by dementia, conduct research, and influence policy, must be included in discussions about supportive care and the priorities for research in this area.

The purpose of this study was to partner with stakeholders to identify gaps in care and from this list, identify priorities for dementia care research. A second purpose was to describe the priorities for dementia care research by stakeholder group and examine for differences by group. To accomplish this, we conducted a survey to capture multiple perspectives, importantly PLWD, family caregivers to PLWD, and health/social care professionals who serve families affected by ADRD. This research was conducted in partnership with a Stakeholder Advisory Council (SAC).

## Methods

To identify priority dementia care research topics, surveys about the importance of research topics identified by the SAC were administered to community members impacted by dementia. A detailed description of the methods we applied follows. This project was submitted to the Institutional Review Board and deemed to be exempt (HSC20180659N). All SAC members provided verbal consent for their participation in identifying topics for research that would serve as survey items and in helping to identify priorities. Participants who completed the surveys to rank the survey items were provided with an information sheet about the survey, which was completed electronically or through an interview conducted on Zoom.

### Partnership with a stakeholder advisory council

The SAC was convened in November 2018 and continues to meet monthly as of March 2021 to address research topics relevant to patient/family-centered dementia care. The SAC consists of 15 members, including 2 PLWD, 4 family caregivers, 7 health/social care professionals, and 2 researchers. Professional members include geriatricians, a nurse with expertise in palliative care, social service program providers, and a member from the faith community. The SAC was co-led by the Principal Investigator for this research (CW), in partnership with MF, who lives with dementia. Co-chairs of the SAC consulted on agenda items each month. Monthly meetings lasted for one to one and a half hours each, with an average of 10 members in attendance. With the beginning of the pandemic and need for social distancing, the SAC transitioned to virtual zoom meetings as of March 2020. SAC meetings were designed to build capacity among members to participate in patient-centered outcomes research. Specifically, we reviewed principles of ethical conduct, discussed patient-centered outcomes research with a focus on supportive care, stakeholder engagement in research, and the role of the SAC in identifying priorities for dementia care research. After participating in generating the list of dementia care research topics, SAC members helped to reduce the list by removing redundant items and those that could not be addressed by research, refining the data collection instrument, participating in recruitment as well as data collection, and providing their interpretation to the results. Additional information about the SAC and the experience of participating in this council are described in Masoud et al. [12].

### Identification of topics for research

Identifying priorities for dementia care research was an iterative process. SAC members described gaps in care from their different perspectives and potential areas of research relevant to these topics, with ethnographic notes taken at each monthly SAC meeting with members’ consent. SAC members were encouraged to discuss dementia-related research topics with others in their personal networks, documenting notable topics to share at the SAC meetings. Through our web-based portal, questions were posted about gaps in care and research topics to address these gaps. From the postings on the portal from the SAC and the wider network, topics for research were also collected. Discussions took place at the monthly meetings around these different topics. From the meeting notes, research topics were extracted and compiled into a list, which was shared with the SAC. An in-person symposium was held in June 2019, with a focus on stakeholder engagement in research. Through small group discussions, led by SAC members, research topics around dementia care were discussed and added to the list. From these different sources, a long list of 86 questions/topics was generated.

### Preparation of survey for prioritization

**Fig. 1:**
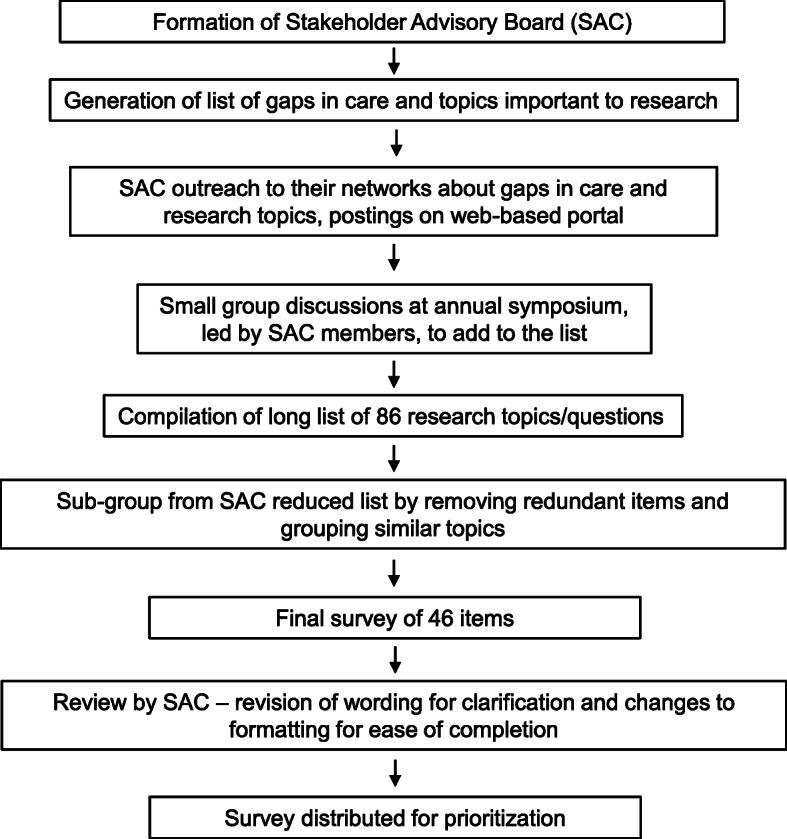
Overview of Process to Develop Survey of Topics Important to Dementia Care Research

A sub-group of SAC members, consisting of 2 researchers, a family caregiver, and 2 clinicians, met over 3 meetings to review the long list. Common ideas were merged, duplicates were removed, and wording was revised for clarity. An underlying principle in designing the survey instrument was to ensure that it would be accessible to all stakeholders, including PLWD. We followed the ‘dementia friendly’ design principles recommended by Morbey et al. [13] in their project of developing a core outcome set for evaluating interventions for PLWD. The SAC was also consulted on the design of the survey instrument. The survey instrument included the use of ‘plain language’, enlarged font, contrasting font and background color, and application of alternating background colors for each question on the survey collection tool. Response options were also abbreviated to 3 response alternatives about the importance of the question, as compared with typically used 5-point Likert scales, to limit decision burden among PLWD. The shortened list was presented to the SAC during a monthly meeting for their review, with minor changes recommended. Edits included changing the items to topics rather than questions, as they found that they were trying to answer each question rather than rate it for importance, as well as design and wording changes to make the instrument more suited for families impacted by dementia. These changes were integrated into the survey instrument and reviewed again by the SAC prior to distribution in August 2020. Figure 1 summarizes this process.

### Sample and data collection

Those eligible to complete the survey were individuals who were personally or professionally impacted by dementia. This included PLWD, family caregivers, and professionals who worked directly or indirectly with families affected by dementia. A snowball sampling technique was used to recruit participants, wherein members of the SAC were asked to distribute the survey to potentially eligible individuals in their respective communities. Community members who administered survey questionnaires were trained to assent PLWD, including processes to attain verbal assent, how to recognize signs that an individual withdrew assent, and how to identify when a participant’s level of cognitive functioning may detrimentally affect their ability to provide meaningful survey responses [14].

**Fig. 2:**
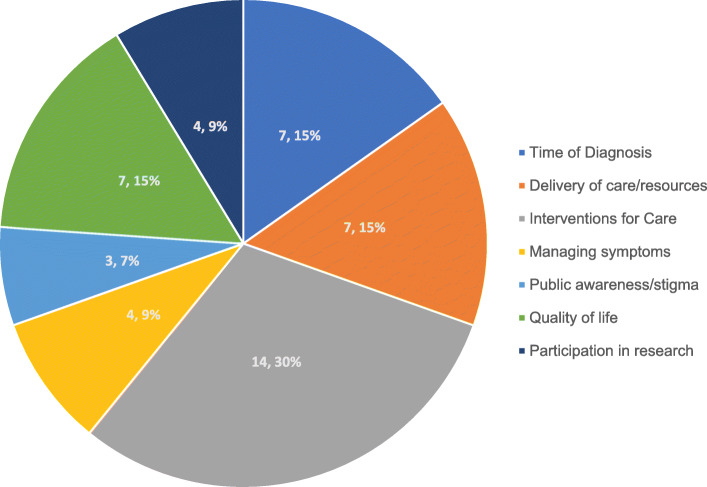
Research Topics (n, %) by Overarching Themes

Surveys were completed both through an electronic link to the Qualtrics survey and ‘face-to-face’ via Zoom. Face-to-face surveys were offered to PLWD and were administered by community members and members of the research team. Participants were asked to rate the importance of each research topic area in the final list of priority research topics consisting of 46 separate topics, grouped into 7 overarching research domains (Fig. 2).

### Measurement

Participants were asked to identify as a PLWD, family caregiver, health or social care provider, researcher, or other; those selecting the “other” option were asked to write in their role. We also collected data about participants’ ethnicity and gender. Survey items were listed as research topics (e.g., How to manage, anxiety, fear, and other emotions related to a diagnosis of dementia). Participants could respond that items were “Not so important,” “Important,” or “Very important.” This wording was selected because all items were considered to be important given that they were identified by the SAC and other stakeholders, and thus there was no option to indicate a topic as being ‘unimportant’, but instructions indicated to think about the relative importance each particular item held for them.

### Analysis

Survey data were analyzed using descriptive and bivariate statistics. We examined the distributions of the responses to the research topics using frequencies and proportions of responses in rating the topics. We also examined those items rated as “Very Important”, stratified by stakeholder group (PLWD, family caregiver, health/social care professional which also included ‘other’). To summarize findings, we present results from the five items with the highest proportion of “Very important” and the 5 items with the lowest proportion of “Very important” for each subgroup. After examining expected frequencies of responses for each sub-group according to importance ratings, we completed bivariate analyses. For those responses wherein expected frequencies were < 5, we applied a Fisher’s exact test. For all other bivariate comparisons, we used a Pearson Chi-squared statistic. Differences with a *p*-value of < 0.05 were considered to be statistically significant in the distribution of responses by subgroup. Analyses were completed in Stata 15.1.

## Results

### Participant characteristics

A total of 186 participants completed the survey from August through October 2020, including 23 (12.4%) PLWD, 101 (54.3%) family caregivers, and 62 (33.3%) classified as health/social care professionals/other. Of this latter group, the majority (*n* = 41, 66.1%) self-identified as health and social care professionals, 4 healthcare students, and 17 as “other”, which included paid caregivers, researchers, and elder lawyers, among others. Participants identified as non-Hispanic White (50%), Hispanic (35%), Black/African American (8.3%), and other race (6.7%). Three-quarters were female (75.6%) and 24.4% were male. See Table 1 for demographic characteristics by stakeholder group.

**Table 1:** Participant Characteristics by Stakeholder Group

	PLWD (*n* = 23)	Caregiver (*n* = 101)	HCP (*n* = 62)
Female, n (%)	12 (52.2)	74 (73.3)	49 (79.0)
Race/Ethnicity^a^, n (%)			
White	15 (65.2)	47 (49.0)	27 (50.0)
Non-White Hispanic	6 (26.1)	42 (43.8)	15 (27.8)
Other	1 (4.3)	7 (7.3)	12 (22.2)

*PLWD* Person Living with Dementia, *HCP* health and social care professionals, researchers, other

^a^missing data on race/ethnicity

### Priority areas

Supplemental Table 1 shows the 46 topics for research included in the final list and the percentage for each topic selected as “Very Important” overall and by stakeholder group (PLWD, family caregiver, and health and social care professional/other). There were 10 research topics that were selected by two-thirds or more of the sample. Three of these topics fell under the theme of research topics related to the time of diagnosis, and included the resources and support that families need at the time of diagnosis (76%), the benefits of an early diagnosis (68%), and how to improve the process of a timely diagnosis (68%). Intervention topics rated as “Very Important” included how to support PLWD to remain in their own homes (73%), how to support families to provide care as the dementia progresses (71%), non-pharmacological interventions to manage behavioral symptoms (69%), and how to support the emotional health of PLWD and their families (68%). Other research topics rated as “Very Important” included interventions to support the dignity of PLWD (70%) and how we can respect individual choices of quality vs. prolonging life (66%). Finally, research that identifies the skills and knowledge needed by the health care support to support PLWD and their families was selected as “Very Important” by 66% of the sample. There were few significant differences across the distribution of frequencies by stakeholder group. Notable significant differences related to special populations (rural areas, those living alone, and different cultures) which were selected as “Very Important” by a higher proportion of health and social care professionals compared with other stakeholder groups. Relative to other stakeholder groups, a higher proportion of PLWD selected research to support individuals with younger onset dementia as well as ways for support when PLWD are unable to make decisions about health and care.

#### Highest priority topics according to stakeholder group

Among PLWD, research focused on support for continuing to live in their own homes was ranked as the highest priority, rated by 91.3% as “Very Important” (Table 2). Among other priority topics were research around advance care planning (78.3%) and respecting individual choices (82.6%). PLWD also prioritized research that identified the benefits of an early diagnosis (73.9%), evidence about information and resources to support families following a diagnosis (73.9%), as well as support for managing the emotional consequences of a diagnosis of dementia (73.9%). The majority (78.2%) also rated the need for research that identifies the skills and knowledge health care professionals need to communication with families as “Very Important”.

**Table 2:** Top 5 “Very Important” Priorities by Stakeholder Group

PLWD (*n* = 23)		Caregiver (*n* = 101)		HCP (*n* = 62)	
Priority	%	Priority	%	Priority	%
**Services and supports that are needed to help people living with dementia continue living in their own homes.**	91.3	Understand the benefits of cognitive stimulation activities such as games and crosswords in delaying the onset or slowing the progression of dementia.	76.3	**Information and resources needed to help individuals and their families following a diagnosis of dementia.**	78.6
Understand how to respect individual choices regarding quality of life versus prolonging life.	82.6	Effective approaches apart from medication to manage behavioral symptoms.	74.7	How a diagnosis of dementia affects the relationships between a person living with dementia and their families.	72.7
Identify ways to support people living with dementia and their families to make advance care plans early in the diagnosis.	78.3	**Information and resources needed to help individuals and their families following a diagnosis of dementia.**	74.2	**How an early diagnosis benefits the person with the diagnosis and their family.**	71.4
Identify the skills and knowledge that the healthcare team needs to provide better care for people living with dementia and their families.	78.3	Identify the care and support services that are needed to support the dignity of people living with dementia.	73.6	How the process of receiving a diagnosis of dementia can be more personalized for the individual and the family.	70.9
**Information and resources needed to help individuals and their families following a diagnosis of dementia.**	73.9	**Services and supports that are needed to help people living with dementia continue living in their own homes.**	72.0	Identify how to support people living with dementia and their families who are vulnerable and at higher risk for poor health outcomes.	70.6
**How an early diagnosis benefits the person with the diagnosis and their family.**	73.9	–			
How to manage anxiety, fear, and other emotions related to a diagnosis of dementia.	73.9				
Ways to support communication and decisions about medications and care among people living with dementia, their families, and health care providers.	73.9				
Ways to support people living with dementia who are unable to make decisions about their health and care.	73.9	–			

Overlapping priorities among groups are bolded

*PLWD* Person Living with Dementia), *HCP* health and social care professionals, researchers, other

High priority research areas for family caregivers included interventions to slow cognitive decline (76.3%) as well as non-pharmacological approaches to manage behavioral symptoms (74.7%). Family caregivers also prioritized evidence for resources and support for families following a diagnosis (74.2%) and support that would allow PLWD to continue living in their own homes (72%).

Priority research topics for health care professionals were focused on the time of diagnosis including the benefits of an early diagnosis (71.4%), how best to provide the diagnosis (70.9%), and the supports needed following a diagnosis (78.6%). Priorities common across all stakeholder groups was the importance of research on how best to support families following a diagnosis of dementia. Furthermore, both PLWD and family caregivers prioritized effective interventions to support the PLWD to continue living in their own homes.

#### Lowest priority topics according to stakeholder group

There were more commonalities than differences across stakeholder groups in research topics rated as low priority based on the proportion of participants rating them as “Very Important” (Table 3). For example, across stakeholder groups, examining how adult day programs support quality of life was given low priority. Identifying work or volunteer opportunities for family caregivers, drawing on what they have learned from their role was scored as “Very Important” by around one-third of PLWD (34.7%), family caregivers. (32.6%) and health/social care professionals (39.2%). A low percentage of PLWD selected culture-specific approaches to supporting at-risk communities (31.8%), research about interventions for financial support (34.8%), and interventions for people with dementia who live alone (41%) as “Very Important”. Identifying effective strategies to engage PLWD and family caregivers in research was also rated as a lower priority by both family caregivers (37.8%) and health/social care professionals (44%).

**Table 3:** Bottom 5 “Very Important” Priorities by Stakeholder Group

PLWD (*n* = 23)		Caregiver (*n* = 101)		HCP (*n* = 62)	
Priority	%	Priority	%	Priority	%
Identify best culture-specific approaches for educating and supporting at-risk communities	31.8	Tracking and supporting statistics on dementia and caregiving at a local level.	31.1	**Identify work or volunteer opportunities for family caregivers to use their skills and knowledge from their caregiving experience.**	39.2
Identifying effective ways to help people living with dementia and their families obtain financial support.	34.8	**Identify the healthcare and support services that are needed for people with younger onset dementia and their families.**	31.5	Understand how trust among healthcare providers and people living with dementia and their families affects health outcomes.	39.2
**Identifying how adult day programs affect the health and quality of life for people living with dementia and their families.**	34.8	**Identify work or volunteer opportunities for family caregivers to use their skills and knowledge from their caregiving experience.**	32.6	**Identify the healthcare and support services that are needed for people with younger onset dementia and their families.**	41.2
**Identify work or volunteer opportunities for family caregivers to use their skills and knowledge from their caregiving experience.**	34.8	**Identifying how adult day programs affect the health and quality of life for people living with dementia and their families.**	37.2	**Understand ways to enable people living with dementia and their families to actively engage in research.**	44.0
Identify ways to support people living with dementia who live alone.	40.9	**Understand ways to enable people living with dementia and their families to actively engage in research.**	37.8	**Identifying how adult day programs affect the health and quality of life for people living with dementia and their families.**	45.1

Overlapping priorities among groups are bolded

*PLWD* Person Living with Dementia, *HCP* health and social care professionals, researchers, other

#### Comparison of top priorities of PLWD with ratings by other stakeholders

We compared the top priorities selected by PLWD with the percentage of family caregivers and health/social care professionals selecting those research topics as “Very Important” (Table 4). There were no significant differences in the distribution of the responses except for the topic that concerned research to support PLWD who are unable to make decisions about their health and health care. This topic was selected as “Very Important” by 73.9% of PLWD, 53.8% of family caregivers and 56.9% of health and social care professionals (*p* = 0.04). There was a trend for significant differences on research about the services and supports that are needed to help people living with dementia continue living in their own homes, with 91.3% of PLWD selecting this as “Very Important” compared with 72% of family caregivers and 66.7% of health/social care professionals (*p* = 0.07).

**Table 4:** Comparison of Top Priorities of PLWD with Rating as “Very Important” by Other Stakeholders

Priority Research Topic	PLWD (*n* = 23) Percentage	Caregiver (*n* = 101) Percentage	HCP (*n* = 62) Percentage	*p*-value from Fisher’s Exact Test
**Services and supports that are needed to help people living with dementia continue living in their own homes.**	91.3	72.0	66.7	0.07
Understand how to respect individual choices regarding quality of life versus prolonging life.	82.6	61.1	66.0	0.37
Identify the skills and knowledge that the healthcare team needs to provide better care for people living with dementia and their families.	78.3	65.2	62.8	0.74
Identify ways to support people living with dementia and their families to make advance care plans early in the diagnosis.	78.3	62.0	48.0	0.12
**Information and resources needed to help individuals and their families following a diagnosis of dementia.**	73.9	74.2	78.6	0.88
**How an early diagnosis benefits the person with the diagnosis and their family.**	73.9	66.0	71.4	0.66
How to manage anxiety, fear, and other emotions related to a diagnosis of dementia.	73.9	58.3	69.1	0.25
Ways to support communication and decisions about medications and care among people living with dementia, their families, and health care providers.	73.9	58.1	52.9	0.31
Ways to support people living with dementia who are unable to make decisions about their health and care.	73.9	53.8	56.9	0.04

Bolded text represents research topics among top priorities for PLWD and at least 1 other stakeholder group

*PLWD* Person Living with Dementia, *HCP* health and social care professionals, researchers, other

## Discussion

This paper reports on the priority areas for dementia care research identified by stakeholders. To add to the science of dementia-specific supportive care, it is important that the voices of all stakeholders are included. To this end, SAC members as well as the larger network accessed through the symposium, webinars, and the web portal contributed, in an iterative fashion, to identifying and prioritizing topics for dementia care research. Findings are intended to inform those who conduct research and those who fund research about which research topics stakeholders believe are most important and thus have greatest potential to improve the quality of life among PLWD and their families.

The findings from this project are consistent with a study conducted by Bethell et al. [15] to identify dementia research priorities among stakeholders in Canada. They conducted a workshop with stakeholders for final prioritization of their short list of 23 questions. Our initial plan was also to discuss the prioritization of the topics for research at an in-person symposium, but we needed to transition to an online survey related to the COVID-19 pandemic. Despite the different processes, there are consistent themes among the priorities for dementia care research, including supports and services after diagnosis, non-pharmacological management of behavioral symptoms, dementia-related knowledge and skills needed by health-care providers, and care that will support the dignity of the PLWD. The James Lind Alliance also conducted a dementia priority setting partnership to identify priorities related to prevention, diagnosis, treatment, and care related to dementia [16]. Similar priorities include the impact of an early diagnosis, supports to continue living at home, as well as a focus on palliative and end of life care. And consistent across all three studies from three different countries is the need for further community support and education for families living with dementia.

We examined the high and low priority topics by stakeholder group. PLWD and family caregivers bring their unique perspectives, drawn from their lived experiences, to identify gaps in care and topics for research. Although there were few significant differences by stakeholder group across the 46 topics for research, there were differences in the ordering of priorities, with PLWD and family caregivers selecting items around diagnosis, cognitive interventions, and the emotional consequences of the diagnosis among their top priorities, while health and social care professional priorities included the process of giving the diagnosis and how a diagnosis impacts the relationship for the PLWD. One reason for these differences may be that professionals are more likely to encounter families who struggle to get a diagnosis and who experience relationship challenges, even if these issues may not affect all families living with dementia.

Topics related to diagnosis and the time following diagnosis were among the priority topics for research across stakeholder groups, including strategies around a timely diagnosis. Challenges with receiving a timely diagnosis may be related to the ongoing beliefs and understanding about aging and cognitive decline. Surprisingly, in a survey conducted by Alzheimer’s Disease International with almost 70,000 participants in 155 countries, 62% of health care professionals still believe that dementia is part of normal aging [17]. Participants within the SAC meetings discussed the gaps in care around diagnosis, with little guidance on what the diagnosis even means and where to go for information, education, and resources. A recent scoping review highlights the limited support for family caregivers during this period of transition into the caregiving role, with their needs following diagnosis including knowledge and information about the progression of the disease, emotional and psychological support, and assistance with care planning [18]. The review identified only 4 interventions tailored to the period surrounding the diagnosis. This suggests an important focus for research, with interventions that could proactively improve long-term health outcomes for family caregivers. Furthermore, interventions aimed at those in early stage of dementia, including information about the progression of the condition and support for advance care planning, identified as a priority by PLWD, could enhance dignity and their quality of life.

Within the priority topics, there were topics for which there are already available evidence-based interventions. In a systematic review, Gitlin and colleagues reported that over 200 interventions have been tested for supporting family caregivers, but few have been translated into practice, remaining inaccessible to most of the more than 16 million family caregivers of PLWD in the US [19]. For example, there are programs with good evidence from randomized controlled trials to help family caregivers in managing the behavioral challenges associated with dementia such as the Tailored Activities Program [20, 21], the Care of Persons with Dementia in their Environments [22], and the Savvy Caregiver Program [23, 24] to name a few, yet most family caregivers do not have access to these programs.

There are currently over 50 million people worldwide with a diagnosis of dementia, projected to increase to 152 million by 2050 [25]. There is an urgent need to address this growing crisis, delivering patient- and family-centered care. It is critical that we study what matters most to these individuals and families impacted by dementia, including translational research that disseminates evidence-based interventions, so they are accessible to the community of PLWD and family caregivers. Crowe et al. [10] reported on the continuing mismatch between what patients, families, and clinicians rank as important topics for research and the research that is actually conducted. In using the data from the James Lind Priority Setting Partnerships and comparing what topics were selected as priorities by stakeholder groups with a random selection of clinical trials, they reported marked differences, with drug studies accounting for the majority of commercial and non-commercial trials, while they accounted for only a small proportion of the priorities selected by the stakeholder groups.

Addressing this mismatch necessitates an increased focus of engagement of PLWD and family caregivers in our research, beyond setting priorities, to functioning as active members of the research team. Yet, results from this project show that engagement in research was rated as “Very Important” by only 41% of participants and was not significantly different by stakeholder group. This result requires further investigation, including how stakeholders perceive ADRD research. Despite this growing emphasis in including stakeholders in all phases of the research, there are still few examples of this being operationalized within research settings. This resistance often comes from researchers and research institutions, with resistance related to the added time to the research to build and maintain trusting relationships [26, 27]. There are several models that can serve as examples of including PLWD and family caregivers in co-producing research and ensuring that research is studying what is relevant to families [4, 28].

### Limitations

When interpreting the results, the limitations of this study should be considered. Participants who completed the survey were from an urban center and thus do not necessarily reflect the priorities for research among those in rural areas. Participants were recruited through email and the survey was completed electronically or through Zoom so may not reflect those without access to technology, particularly underserved populations. We also acknowledge that our sampling technique which included members of the SAC reaching out to their networks to participate in the priority-setting could have led to bias as participants may not be independent of one another. This was, however, just one technique used for recruitment and the diversity of the SAC may have contributed to more diversity within the sample. We included only those with early-stage dementia, so the priorities might not be the same for those in later stages of dementia. Further, surveys to PLWD were interview-administered over Zoom to ensure they were accessible compared with online completion by other participants and this could lead to potential differences in responses. We utilized a 3-point rating scale recommended from the work of Morbey et al. [13] to ensure that the survey was accessible to PLWD. This most likely limited the variability in rating the different topics. Despite this, our findings are consistent with previous priority-setting projects among dementia stakeholders [15, 16]. The low representation of African American/Black individuals (8.3%) impacts on conclusions we can draw for this group. For example, specific needs of African American caregivers to PLWD may be reflected in differences in their priorities for dementia care research [29]. Given the disparities in dementia and dementia care for this racial group [30], there is an urgent need to ensure that future research captures their priorities for dementia care research.

## Conclusion

This project draws on the strengths of its multi-stakeholder perspective and the aim of generating patient-centered outcomes research questions was accomplished. These results offer directions for researchers and funding agencies across the trajectory of the condition, including diagnosis, resources and supports needed as the dementia progresses, including supports needed to support the family and to help keep the PLWD in the community. These findings lend support to the importance of recent policy efforts to adopt evidence-based models of support for families impacted by dementia, such as the RAISE (Recognize, Assist, Include, Support, and Engage) Family Caregiver Act [31]. This project was focused on supportive care for families impacted by dementia and the priorities identified here do not preclude the important need for continued research that investigates other areas of importance to dementia, including mechanisms of the disease, risk factors, prevention, and disease-modifying therapies.

## Supplementary Information

**Additional file 1 **: **Supplemental Table 1**. Percentage Selecting Research Topic as “Very Important” Overall and by Stakeholder Group.

## Data Availability

All data generated or analyzed during this study are included in this published article [and its supplementary information file].

## References

[1] Frank L, Morton SC, Guise JM, Jull J, Concannon TW, Tugwell P (2020). Engaging patients and other non-researchers in health research: Defining research engagement. Journal of General Internal Medicine.

[2] Woolf SH, Zimmerman E, Haley A, Krist AH (2016). Authentic engagement of patients and communities can transform research, practice, and policy. Health Affairs.

[3] Frank L, Shubeck E, Maslow K, Epstein-Lubow G (2020). Meta-issues: On writing scientific manuscripts with a stakeholder group of persons living with dementia. The American Journal of Geriatric Psychiatry.

[4] Harrison J, Maslow K, Tambor E, Phillips L, Frank L, Herndon L, Epstein-Lubow G (2020). Engaging stakeholders in the design and conduct of embedded pragmatic clinical trials for Alzheimer's disease and Alzheimer's disease-related dementias. Journal of the American Geriatrics Society.

[5] Kirwan JR, de Wit M, Frank L, Haywood KL, Salek S, Brace-McDonnell S, Lyddiatt A, Barbic SP, Alonso J, Guillemin F, Bartlett SJ (2017). Emerging guidelines for patient engagement in research. Value in Health.

[6] Rukes K, Fowler N (2020). Engaging persons living with dementia in the research process: Best practice considerations from a national dementia meeting. The American Journal of Geriatric Psychiatry.

[7] Bethell J, Commisso E, Rostad HM, Puts M, Babineau J, Grinbergs-Saull A, Wighton MB, Hammel J, Doyle E, Nadeau S, McGilton KS (2018). Patient engagement in research related to dementia: A scoping review. Dementia (London).

[8] Alzheimer's Disease Facts and Figures (2020). Alzheimers. Dementia.

[9] Crumby AS, Holmes ER, Rosenthal M (2018). Patient centered research to improve community involvement (PaRTICIpate) in diabetes self-management: A conference series for developing collaborations between researchers, stakeholders, and patients. J Patient Rep Outcomes.

[10] Crowe S, Fenton M, Hall M, Cowan K, Chalmers I (2015). Patients’, clinicians’ and the research communities’ priorities for treatment research: There is an important mismatch. Research Involvement and Engagement.

[11] Hao Z, Ruggiano N (2020). Family-centeredness in dementia care: What is the evidence?. Social Work in Health Care.

[12] Masoud, S. S., Glassner, A., Patel, N., Mendoza, M., James, D., Rivette, S., et al. (2021). Engagement with a diverse stakeholder advisory council for research in dementia care. *Research Involvement and Engagement*. (in press) 10.1186/s40900-021-00297-8 PMC8300992 34301338

[13] Morbey H, Harding AJE, Swarbrick C, Ahmed F, Elvish R, Keady J, Williamson PR, Reilly ST (2019). Involving people living with dementia in research: An accessible modified Delphi survey for core outcome set development. Trials.

[14] Hellström I, Nolan M, Nordenfelt L, Lundh U (2007). Ethical and methodological issues in interviewing persons with dementia. Nursing Ethics.

[15] Bethell J, Pringle D, Chambers LW, Cohen C, Commisso E, Cowan K, Fehr P, Laupacis A, Szeto P, McGilton KS (2018). Patient and public involvement in identifying dementia research priorities. Journal of the American Geriatrics Society.

[16] Kelly S, Lafortune L, Hart N, Cowan K, Fenton M, Brayne C, Dementia Priority Setting Partnership (2015). Dementia priority setting partnership with the James Lind Alliance: Using patient and public involvement and the evidence base to inform the research agenda. Age and Ageing.

[17] Alzheimer's Disease International. World Alzheimer Report (2019). Attitudes to dementia: a global survey.

[18] Lee K, Puga F, Pickering CEZ, Masoud SS, White CL (2019). Transitioning into the caregiver role following a diagnosis of Alzheimer’s disease or related dementia: A scoping review. International Journal of Nursing Studies.

[19] Gitlin LN, Marx K, Stanley IH, Hodgson N (2015). Translating evidence-based dementia caregiving interventions into practice: State-of-the-science and next steps. Gerontologist.

[20] Gitlin LN, Winter L, Burke J, Chernett N, Dennis MP, Hauck WW (2008). Tailored activities to manage neuropsychiatric behaviors in persons with dementia and reduce caregiver burden: A randomized pilot study. The American Journal of Geriatric Psychiatry.

[21] Gitlin LN, Winter L, Dennis MP, Hodgson N, Hauck WW (2010). Targeting and managing behavioral symptoms in individuals with dementia: A randomized trial of a nonpharmacological intervention. Journal of the American Geriatrics Society.

[22] Gitlin LN, Winter L, Dennis MP, Hodgson N, Hauck WW (2010). A biobehavioral home-based intervention and the well-being of patients with dementia and their caregivers: The COPE randomized trial. JAMA.

[23] Hepburn K, Lewis M, Tornatore J, Sherman CW, Bremer KL (2007). The savvy caregiver program: The demonstrated effectiveness of a transportable dementia caregiver psychoeducation program. Journal of Gerontological Nursing.

[24] Hepburn KW, Lewis M, Sherman CW, Tornatore J (2003). The savvy caregiver program: Developing and testing a transportable dementia family caregiver training program. Gerontologist.

[25] World PC, Report A (2018). The state of the art of dementia research: New frontiers.

[26] Heckert A, Forsythe LP, Carman KL, Frank L, Hemphill R, Elstad EA, Esmail L, Lesch JK (2020). Researchers, patients, and other stakeholders’ perspectives on challenges to and strategies for engagement. Research Involvement and Engagement.

[27] Yarborough M, Edwards K, Espinoza P, Geller G, Sarwal A, Sharp R, Spicer P (2013). Relationships hold the key to trustworthy and productive translational science: Recommendations for expanding community engagement in biomedical research. Clinical and Translational Science.

[28] Swarbrick CM, Doors O, Educate DK, Keady J (2019). Visioning change: Co-producing a model of involvement and engagement in research (innovative practice). Dementia (London).

[29] Samson ZB, Parker M, Dye C, Hepburn K (2016). Experiences and learning needs of African American family dementia caregivers. American Journal of Alzheimer's Disease and Other Dementias.

[30] Alzheimer’s Disease Facts and Figures (2021). Alzheimers. Dement.

[31] H.R.3759 - RAISE Family Caregivers Act (2017–2018). https://www.congress.gov/bill/115th-congress/house-bill/3759/text. Accessed 10 May 2021.

