# Supplementary material for: Engaging multi-stakeholder perspectives to identify dementia care research priorities
Source: J Patient Rep Outcomes. 2021 Jun 22;5:46. doi: 10.1186/s41687-021-00325-x (PMC8218276; doi:10.1186/s41687-021-00325-x)
Supplement: Supplementary file 1 — Additional file 1 : Supplemental Table 1. Percentage Selecting Research Topic as “Very Important” Overall and by Stakeholder Group. [file 41687_2021_325_MOESM1_ESM.docx]

Supplemental Table 1. Percentage Selecting Research Topic as “Very Important” Overall and by Stakeholder Group

| Research Topic | Overall | PLWD  (n=23) | Family Caregiver (n=101) | HCP  (n=62) | p-value** |
| --- | --- | --- | --- | --- | --- |
| **Research Topics Related to the Time of Diagnosis** |  |  |  |  |  |
| Information and resources needed to help individuals and their families following a diagnosis of dementia. | **75.6*** | 73.9 | 74.2 | 78.6 | 0.88 |
| How the process of receiving a diagnosis of dementia can be more personalized for the individual and the family. | 61.5 | 52.2 | 58.3 | 70.9 | 0.06 |
| How an early diagnosis benefits the person with the diagnosis and their family. | **68.2** | 73.9 | 65.0 | 71.4 | 0.66 |
| How to support accepting a diagnosis of dementia for people living with dementia and their families who may be in denial. | 57.8 | 47.8 | 54.7 | 67.3 | 0.30 |
| How to manage anxiety, fear, and other emotions related to a diagnosis of dementia. | 63.8 | 73.9 | 58.3 | 69.1 | 0.25 |
| How to improve the process for a more timely and accurate diagnosis of dementia. | **68.2** | 60.9 | 69.2 | 69.6 | 0.44 |
| How a diagnosis of dementia affects the relationships between a person living with dementia and their families. | 65.1 | 56.5 | 62.9 | 72.7 | 0.37 |
| **Research Topics Related to Resources for Managing Dementia and its Consequences** |  |  |  |  |  |
| Identifying effective ways to help people living with dementia and their families obtain financial support. | 47.0 | 34.8 | 47.9 | 51.0 | 0.52 |
| Ways to support people living with dementia and their families to navigate and understand Medicare, Medicaid, or other insurance programs. | 51.2 | 52.2 | 52.1 | 49.0 | 0.86 |
| Determine the most effective education models for people living with dementia and their families. | 50.9 | 50.0 | 47.9 | 56.9 | 0.83 |
| Identifying barriers that might prevent people living with dementia and their families from seeking the help they need. | 50.3 | 60.9 | 42.1 | 60.8 | 0.07 |
| Identifying how adult day programs affect the health and quality of life for people living with dementia and their families.^1^ | 39.3 | 34.8 | 37.2 | 45.1 | 0.54 |
| Services and supports that are needed to help people living with dementia continue living in their own homes. | **73.1** | 91.3 | 72.0 | 66.7 | 0.07 |
| How to ensure that people living with dementia and their families in rural areas have the resources they need.^1^ | 41.9 | 27.3 | 37.2 | 56.9 | 0.02 |
| **Research Topics Related to Care Interventions/Person-Centered Care** |  |  |  |  |  |
| How to support families to provide care for a person living with dementia as the dementia progresses. | **71.3** | 74.0 | 76.3 | 60.8 | 0.17 |
| How to help families, friends, and health care providers work together to support people living with dementia. | 59.3 | 60.9 | 53.8 | 68.6 | 0.08 |
| Ways to support communication and decisions about medications and care among people living with dementia, their families, and health care providers. | 58.7 | 73.9 | 58.1 | 52.9 | 0.31 |
| Identify the skills and knowledge that the healthcare team needs to provide better care for people living with dementia and their families. | **66.3** | 78.3 | 65.2 | 62.8 | 0.74 |
| Understand how trust among healthcare providers and people living with dementia and their families affects health outcomes. | 51.5 | 65.2 | 54.8 | 39.2 | 0.09 |
| Identify ways to support people living with dementia and their families to manage complex medical care. | 65.3 | 82.6 | 65.6 | 56.9 | 0.16 |
| Identify ways to support people living with dementia and their families to make advance care plans early in the diagnosis. | 60.0 | 78.3 | 62.0 | 48.0 | 0.12 |
| Ways to support people living with dementia who are unable to make decisions about their health and care. | 57.5 | 73.9 | 53.8 | 56.9 | 0.04 |
| Identify work or volunteer opportunities for family caregivers to use their skills and knowledge from their caregiving experience. ^1^ | 34.9 | 34.8 | 32.6 | 39.2 | 0.62 |
| Identify the healthcare and support services that are needed for people with younger onset dementia and their families. ^1^ | 36.4 | 45.5 | 31.5 | 41.2 | 0.01 |
| Identify ways to support people living with dementia who live alone. ^1^ | 49.7 | 40.9 | 42.4 | 66.7 | 0.002 |
| Identify the care and support services that are needed to support the dignity of people living with dementia. | **69.7** | 65.2 | 73.6 | 64.7 | 0.22 |
| Identify how to support people living with dementia and their families who are vulnerable and at higher risk for poor health outcomes. | 58.5 | 69.6 | 48.9 | 70.6 | 0.07 |
| Identify best culture-specific approaches for educating and supporting at-risk communities. | 52.2 | 31.8 | 47.8 | 68.6 | <0.001 |
| **Research Topics Related to Managing Symptoms of Dementia** |  |  |  |  |  |
| Effective approaches apart from medication to manage behavioral symptoms. | **69.1** | 52.2 | 74.7 | 66.7 | 0.17 |
| Understand how medications, supplements, and the environment might affect symptoms of dementia. | 64.9 | 67.0 | 67.0 | 62.8 | 0.88 |
| Understand the benefits of cognitive stimulation activities such as games and crosswords in delaying the onset or slowing the progression of dementia. | 64.4 | 54.6 | 68.1 | 62.0 | 0.51 |
| Understand the impact of the food system including pesticides and nutrition in the development of dementia. | 53.7 | 60.9 | 51.1 | 54.9 | 0.95 |
| **Research Topics Related to Public Awareness/Stigma** |  |  |  |  |  |
| Identify ways that communities can support people to live well with dementia. | 53.4 | 59.1 | 46.2 | 64.0 | 0.16 |
| Understand the impact of stigma on the well-being and quality of life of people living with dementia and their families. | 50.3 | 54.6 | 48.4 | 52.0 | 0.87 |
| Determine effective ways to raise awareness and motivate action to address and reduce stigma. | 55.2 | 63.6 | 50.6 | 60.0 | 0.68 |
| **Research Topics Related to Ways to Quality of Life** |  |  |  |  |  |
| Understand the impact of social interactions on the quality of life for people living with dementia and their families. | 59.5 | 65.2 | 54.4 | 66.0 | 0.48 |
| Understand ways to support the mental and emotional health of people living with dementia and their families. | **67.5** | 65.2 | 67.8 | 68.0 | 1.00 |
| Understand ways to help people living with dementia and their families keep hope while a cure is being researched. | 50.9 | 52.2 | 50.0 | 52.0 | 0.87 |
| Understand the most effective ways to design support groups to improve quality of life for people living with dementia and their families. | 53.4 | 56.5 | 47.8 | 62.0 | 0.56 |
| Identify ways to support family caregivers to experience positive aspects in their caregiving role. | 63.2 | 65.2 | 60.0 | 68.0 | 0.27 |
| Understand how people living with dementia and their families use their personal experiences to improve their quality of life. | 50.9 | 56.5 | 48.9 | 52.0 | 0.94 |
| Understand how to respect individual choices regarding quality of life versus prolonging life. | **65.6** | 82.6 | 61.1 | 66.0 | 0.37 |
| **Research Topics Related to Research Participation and Results** |  |  |  |  |  |
| Ways researchers can make results more accessible to people living with dementia and their families. | 53.4 | 69.6 | 52.2 | 48.0 | 0.52 |
| Understand ways to enable people living with dementia and their families to actively engage in research. | 41.7 | 52.2 | 37.8 | 44.0 | 0.77 |
| Understand how researchers can value and respect the unique experiences and spirituality of people living with dementia and their families in their studies. | 54.6 | 59.1 | 50.6 | 60.0 | 0.70 |
| Tracking and reporting statistics on dementia and caregiving at a local level. ^1^ | 39.3 | 56.5 | 31.1 | 46.0 | 0.08 |

PLWD (person living with dementia); HCP (includes health and social care professional, researchers, and other)

*Bolded percentages in Column 2 represent research topics selected as “Very Important” by at least 2/3 of sample

**Comparing percentages by stakeholder group; p-values reported from Pearson Chi Squared analysis
